# French general practitioners’ and patients’ acceptability of a public commitment charter and patient information leaflets targeting unnecessary antibiotic use: a qualitative study

**DOI:** 10.1186/s13756-022-01065-3

**Published:** 2022-02-08

**Authors:** Anaïs Essilini, Gaëlle Le Dref, Aurélie Bocquier, Joëlle Kivits, Adeline Welter, Céline Pulcini, Nathalie Thilly, the AntibioCharte scientific committee

**Affiliations:** 1 Université de Lorraine, APEMAC, F-54000 Nancy, France; 2 Caisse Primaire d’Assurance Maladie du Bas Rhin, 67000 Strasbourg, France; 3 Service de Maladies Infectieuses Et Tropicales, Université de Lorraine, CHRU-Nancy, 54000 Nancy, France; 4 Département Méthodologie, Promotion, Investigation, Université de Lorraine, CHRU-Nancy, 54000 Nancy, France

**Keywords:** Primary care, Antibiotic resistance, General practitioner, Qualitative study, Education, Antimicrobial stewardship, Patient, Intervention, Commitment charter, Non-prescription pad

## Abstract

**Background:**

The ‘AntibioCharte’ randomised controlled study aimed at assessing the impact of a multifaceted antibiotic stewardship intervention targeting French general practitioners with higher-than-average antibiotic use. The intervention included a public commitment charter signed by the general practitioner, a non-prescription pad, and a patient information leaflet.

**Objectives:**

We conducted a qualitative study to evaluate general practitioners’ fidelity in the intervention and its acceptability by patients and general practitioners.

**Methods:**

This investigation was performed in northeastern France from July 2019 to May 2020, among the AntibioCharte intervention group after a 1-year implementation period. General practitioners’ fidelity in the charter was assessed by direct observations; general practitioners’ fidelity in the other tools, and acceptability of both general practitioners and patients were assessed through semi-structured face-to-face individual interviews.

**Results:**

Twenty-seven general practitioners and 14 patients participated. General practitioners’ fidelity varied according to the tool: the charter was clearly displayed in most waiting rooms; the non-prescription pad was used throughout the intervention period by most general practitioners while the leaflet was used by fewer general practitioners. Both general practitioners and patients found the charter’s content and form relevant, but few general practitioners felt themselves publicly engaged. The waiting room may not be appropriate to display the charter as some general practitioners forgot it and patients did not always read the displayed documents. General practitioners appreciated the pad and found that it could help them change their practices. It was perceived as a good tool to educate patients and manage their expectations for antibiotics. Patients appreciated the pad too, especially information on the infections’ symptoms and their duration. Still, some patients feared that it could encourage doctors not to prescribe antibiotics. Unlike general practitioners, who considered the leaflet redundant with the information given during the consultation, patients found it useful to raise awareness on antibiotics’ specificities and risks, and remind them of good practices.

**Conclusions:**

The AntibioCharte intervention was overall well accepted by general practitioners and patients. The non-prescription pad was the best perceived tool.

*Trial registration number* ClinicalTrials.gov: NCT04562571.

**Supplementary Information:**

The online version contains supplementary material available at 10.1186/s13756-022-01065-3.

## Background

Over the last 2 decades, antibiotic resistance has become a major global public health threat [1]. Reducing unnecessary antibiotic use is essential to tackle this [2–5]. Most antibiotics are prescribed in primary care (78% in 2019 in France) [6], especially by general practitioners (GPs) who are responsible for 70% of those prescriptions [7].

Among antibiotic stewardship interventions, many educational interventions targeting prescribers have been studied, with heterogeneous results and a relatively low quality of evidence [8]. As patients may exert pressure on prescribers to get antibiotics, education should target not only prescribers but also patients [9–12]. However, few interventions targeting both of them have been conducted, with contradictory findings [5, 13, 14].

In this context, we conducted a randomised controlled study (AntibioCharte study) to assess the impact of an educational intervention targeting both GPs and patients on antibiotics prescribed by French GPs, as compared with no intervention. The intervention relied on three tools, used together, delivered by the GP to his/her patient, which aimed at engaging GPs, at facilitating the doctor/patient communication and at decreasing patients’ expectations for antibiotics: (1) a charter displaying the public commitment of the GP in promoting antibiotic stewardship (called thereafter ‘charter’), focussing on reducing unnecessary antibiotic use; (2) a non-prescription pad (‘pad’) to be used when an antibiotic is not recommended (e.g. for viral infections); and (3) a patient information leaflet (‘leaflet’) to be used when antibiotics are prescribed.

As part of the secondary objectives of the AntibioCharte study, we evaluated the fidelity of GPs (belonging to the intervention group) regarding the intervention and its acceptability by patients and GPs; this manuscript focuses on these objectives.

## Methods

We performed a qualitative study nested within the randomised controlled study, using observations and semi-structured face-to-face individual interviews. Fidelity evaluated the extent to which the intervention’s tools were used as planned in the daily practice, i.e. for each patient with an infectious disease [15], and the maintenance of their use over the 1-year period [16]. Acceptability evaluated the perceptions of these tools and their interest for antibiotic stewardship by GPs and patients. Fidelity and acceptability were evaluated among GPs and patients of the intervention group 1 year after the intervention started (1st October 2018).

GPs’ fidelity in the intervention was assessed: (1) for the charter, by direct observation by a member of the research team, who visually checked the display of the charter in the practice waiting room; (2) for the pad and the leaflet, by open-ended questions to GPs during the individual interviews. We distinguished a systematic use (for all patients with an infectious disease as intended), an ‘occasional use’ and a ‘non use’, and considered the duration of the use over the year.

GPs’ and patients’ acceptability of the intervention were assessed by open-ended questions during the individual interviews.

This investigation follows the COREQ reporting guidelines (see Additional file 1: Table S1 available as Additional file data) [17].

### Participants and setting

The AntibioCharte study was carried out in Lorraine, a region in the northeast of France, with the support of the regional Health Insurance, the regional health agency and the regional antibiotic stewardship network (AntibioEst). It targeted GPs with higher-than-average antibiotic use in the second quarter of 2017, based on two pay-for-performance indicators routinely used by the National Health Insurance [18] (> 25 antibiotics/100 patients 16–65 years old and without chronic illness and broad-spectrum antibiotics (co-amoxiclav, fluoroquinolones and third and fourth-generation cephalosporins) prescribed in more than 27% of cases).

GPs interviewed for this qualitative study were randomly selected among the 109 GPs included in the intervention group, whether they used one or more of the intervention’s tools or not. Randomisation was stratified by the type of practice (solo or group), with a planned final sample target of 30 GPs. When they initially accepted to participate in the AntibioCharte study, GPs agreed that they could be randomly selected for a qualitative interview 1 year after the start of the intervention.

Patients were recruited among patients of GPs who: (1) participated in this qualitative study; (2) declared having regularly used the tools; and (3) accepted to invite some of their patients to participate or accepted that the research team directly recruits patients in their waiting room. Patients may have been in contact or not with the tools. A final sample of 15 patients was targeted, depending on data saturation.

### Intervention

The intervention was in place from 1st October 2018 to 30th September 2019 and was based on three tools which targeted both GPs and patients with the objective of reducing unnecessary antibiotic use:

1. A public commitment charter promoting antibiotic stewardship, signed by the GP and displayed in the practice waiting room (see Additional file 1: Document S1 available as Additional file data). In this charter, the GP committed him(her)self publicly to prescribe antibiotics only when necessary, e.g., not to prescribe antibiotics for suspected viral infections;
2. A non-prescription pad to be used by the GP during the consultation to educate his/her patient in situations when an antibiotic is not needed, e.g., for viral infections (see Additional file 1: Document S2 available as Additional file data). This document includes the main symptoms of five common viral infections (common cold, flu, sore throat, bronchitis and otitis) and their duration, safety-netting recommendations and key messages promoting antibiotic stewardship (e.g., adverse events of antibiotics, including bacterial resistance);
3. A patient information leaflet to be used by the GP when antibiotics are prescribed, to raise patients’ awareness regarding the specificities of antibiotics (see Additional file 1: Document S3 available as Additional file data). This document, complementary to the prescribing order, includes details on the antibiotic treatment (molecule, dose, and duration), recommendations promoting appropriate use of antibiotics (including discouraging self-medication) and information on adverse events.

The charter and leaflet have been developed and validated by the AntibioCharte scientific committee, based on previous studies using comparable tools [13, 19], and have been pilot-tested among GPs and patients. The pad was developed by the French National Health Insurance in 2015 but is still now largely unknown among GPs [20–22].

### Data collection

Observation of the display of the charter in the waiting room was performed by AE or GLD before the GP’s interview, without the latter being previously informed. The charter was considered either: clearly visible, if displayed in A3 paper size without any (or only few other posters) around; moderately visible, if displayed in A4 or A3 paper size with a lot of other posters around; or hardly visible, if covered by other posters, whatever its size.

To conduct the semi-structured interviews, two interview guides (one for GPs, one for patients) were elaborated by a multidisciplinary team composed of two public health specialists (one junior, one senior) with expertise in qualitative studies (AE, NT), an epistemologist (GLD), a sociologist (JK), and an infectious diseases specialist (CP). For GPs, the guide (see Additional file 1: Document S4 available as Additional file data) explored: (1) their use (e.g., the reported fidelity) and perceptions of the tools (e.g., attractiveness, usefulness, suggestions to improve tools); (2) their perceptions of patients’ acceptability of the tools; (3) the impact of the intervention on their practice. For patients, the guide (see Additional file 1: Document S5 available as Additional file data) explored: (1) their perception of the tools; (2) the impact they perceived on their medical care; and (3) the impact on their views on antibiotics and their individual responsibility regarding antibiotic resistance. The tools were shown to patients before the beginning of the interview to discuss them.

GPs were interviewed from July to October 2019 at their practice; patients were interviewed from January to May 2020 at the GP’s practice or at home. After an oral consent, the interviews were recorded and transcribed. Participation was voluntary and not compensated.

GPs’ sociodemographic and professional characteristics were collected at their inclusion in the AntibioCharte study, and patients’ characteristics at the end of the interview.

### Analysis

Each transcript was analysed independently by GLD and AE. A thematic analysis was conducted using QSR International’s NVivo 11 software. Two analysis grids (one for GPs, one for patients) were designed by GLD and AE and validated by the senior public health and infectious disease specialists. Each theme and subtheme were discussed until a consensus was reached between AE and GLD. Each interview was then coded by AE according to the corresponding analysis grid. Only the themes and subthemes that address the study objectives are presented in the results.

## Results

Out of the 30 contacted GPs, one GP was unavailable during the interview period and could not be interviewed; 29 GPs were interviewed, and 27 interviews were finally included in the analysis as two interviews could not be transcribed due to technical problems with the recordings (mean duration of the interview: 20 ± 10 min). More than half of participants were men, older than 50 years and practiced in a rural area (Table 1). Out of 25 patients invited to participate, 14 agreed to be interviewed (mean duration of the interview: 29 ± 10 min). They were mostly from rural areas (Table 2).

**Table 1:** General practitioners’ characteristics (n = 27)

Characteristics	N	(%)
Age (years)		
< 40	3	(11)
[40–50]	8	(30)
> 50	16	(59)
Sex		
Male	17	(63)
Female	10	(37)
Practice’s location		
Rural	19	(70)
Suburban	3	(11)
Urban	5	(19)
Type of practice		
Solo	16	(59)
Group	11	(41)
Supervisor of general practitioner trainees	8	(30)
Recent^a^ training in infectious diseases	2	(7)
Participation to a group of peers	4	(15)

^a^Any postgraduate training on infectious diseases (continuing medical education) in the year prior to the interview

**Table 2:** Patients’ characteristics (n = 14)

Characteristics	N	(%)
Age (years)		
< 40	3	(21)
[40–50]	1	(7)
[50–60]	4	(29)
[60–70]	1	(7)
≥ 70	5	(36)
Sex		
Male	5	(36)
Female	9	(64)
Educational level^a^		
Primary	1	(7)
Secondary	8	(57)
High-school diploma	2	(14)
> High-school diploma	2	(14)
Occupational status		
Unemployed	1	(7)
Employed	8	(57)
Retired	5	(36)
Personal experience of antibiotic resistance	3	(21)
General practitioner practice’s location		
Rural	11	(79)
Suburban	0	(0)
Urban	3	(21)

^a^One participant did not answer

### GPs’ fidelity in the intervention

At the end of the 1-year intervention period, the charter was displayed in the waiting room in almost all practices (n = 22). It was clearly visible in most cases (n = 16), sometimes only moderately visible (n = 5), and in one case hardly visible.

Most GPs (n = 16) reported a systematic or quasi systematic use of the pad with concerned patients throughout the intervention period. A few (n = 3) reported having used it only at the beginning of the study, corresponding to the cold season when viral infections are more frequent. Other GPs reported either an occasional use (n = 5), or no use at all (n = 3) throughout the year. When GPs used the pad during the consultation, they often took the time to explain it to the concerned patients and less frequently gave it and let patients read it at home.

About half of the GPs (n = 12) reported a systematic or quasi systematic use of the leaflet throughout the intervention period. Others reported having used it just during the first months of the study (n = 8), or no use at all (n = 7).

Finally, about half of the GPs (n = 12) used all three tools throughout the intervention period, and three GPs did not use any tool. GPs found it difficult to use systematically the pad and the leaflet for all concerned patients, as planned in the intervention, and preferred using them as convenient, i.e., when they felt it was useful for the patient understanding.

### GPs’ acceptability of the intervention (Table 3)

**Table 3:** Selection of the most illustrative verbatims from general practitioners’ interviews

Subtheme	Quote	Interview number	Verbatim
Antibiotic stewardship interventions/AntibioCharte	1	GP 21	“What is important, indeed, is to continue to receive any kind of intervention […] on antibiotic prescriptions”
	2	GP 11	“We are always in the first line. So, it’s up to us to be careful.”
	3	GP 2	“You know when you have one [patient] coming, if he/she is just coughing, you know he/she is going to have his/her antibiotics, period! It is not even possible to discuss, it’s not even possible to try to discuss!”
	4	GP 14	“I think it’s useful for most people, the pad is well explained, it details the different infections for which antibiotics should not be prescribed […] it gives explanation [on the non-prescription].”
	5	GP 20	“Yes, I think my statistics [prescription rate of total and broad-spectrum antibiotics] were better last time: antibiotics, [I prescribed] less… it’s thanks to the intervention!”
	6	GP 13	“Generally, I use the antibiotics [very well], and have done so for a very long time, even if my statistics are discordant […] I obtained other data and I use a quarter of what my colleagues use, and that is rather consistent with my practice.”
Commitment charter	7	GP 24	“I think it [the charter] is unnoticed, I don’t think people read much [the posters in the waiting room].”
	8	GP 21	“I must admit about the charter: I don't go in the waiting room. Well, I display it, but then I don't necessarily think about it.”
Non-prescription pad	9	GP 18	The discussion [with patients] is easier with the pad: ‘it [the infection] doesn’t need antibiotics’.”
	10	GP 16	“Again, the pad helped me a lot, it allowed me to sort out two or three complicated situations.”
	11	GP 13	“I had to stop [using the tools], because people… I don’t know what happened in the town… […] they weren’t happy, I had to stop [give the pad and the leaflet] … […] They spread the news, so I had to stop. I didn’t want them to say in the town that…”
	12	GP 19	“It obliges us to be clear in our head. Because if we give the pad [to patients] and think the opposite… it’s not consistent”
Patient information leaflet	13	GP 1	“[…] it's something additional but it's still redundant with the prescription, it's more the information at the bottom [information on adverse events and antibiotic resistance] that seems important to me”

#### AntibioCharte: an intervention well appreciated by GPs

Most GPs welcomed the intervention, which was perceived as a reminder of other antibiotic stewardship actions (e.g., those from the Health Insurance). Even GPs who rarely used the tools approved the principle of the intervention (Quote 1). Indeed, most participants were aware of the relationship between antibiotics’ overprescription in primary care and antibiotic resistance and considered GPs to be the "key players" to tackle this issue (Quote 2). Moreover, many admitted feeling often powerless when faced to patients’ pressure to be prescribed antibiotics (Quote 3).

However, a majority of GPs reported some barriers to use the tools routinely, such as: (1) the time required to explain the pad or the leaflet; (2) the redundancy between what they say to patients during the consultation and the content of the documents; and (3) the practical organisation induced by the tools’ use (e.g., enough space on their desk to put the documents). Half of the GPs wished to continue using the tools after the end of the study (8 all three tools, and 6 the pad only).

Most GPs thought that the intervention and tools were well accepted and useful for patients (Quote 4), although some of them expressed reservations about their effectiveness among some patients (e.g., with a low educational or health literacy level, or reluctant to the non-prescription of antibiotics).

Concerning the impact of the intervention on their prescribing practices, half of the GPs perceived an improvement (Quote 5). The other half considered themselves to prescribe less antibiotics than average, and thus that they have no possibility to further improve their practices (Quote 6). Few participants feared a risk of under-prescription due to antibiotic stewardship interventions like AntibioCharte.

#### The charter: a relevant message but limited effects on patient-GP communication and GPs’ commitment

All GPs who displayed the commitment charter considered the message of the charter to be clear and relevant.

However, almost all GPs were doubtful whether the charter had any effect among patients (Quote 7); some GPs even believed that patients did not read documents displayed in the waiting room. A few GPs thought that the charter was beneficial for patients because it prepared them for a non-prescription of antibiotics and opened the discussion on antibiotic resistance with the doctor.

Few GPs reported feeling themselves publicly engaged by the commitment charter and few others even acknowledged that they had forgotten it (Quote 8).

#### The non-prescription pad: the most valued tool

The pad was the most appreciated tool; the majority of GPs who used it considered that it was a good tool to educate patients. GPs found it helpful to communicate and negotiate with their patients, especially to manage pressure to obtain antibiotics (Quotes 9–10). They also considered it as a reassuring tool that allowed them to justify a non-prescription of antibiotic and to persist in their decision.

Half reported that their patients appreciated the pad (particularly information on symptoms’ durations) and understood it. Few others, particularly those located in rural areas, had experienced hostility from their patients and had sometimes been forced to stop using it (Quote 11). Some GPs thought that the pad could deter patients from coming back a few days later seeking antibiotics.

Many GPs found that the pad could help them change their antibiotic prescribing practices both by reducing unnecessary use and improving the choice of the antibiotic (Quote 5). The information about the possibility of disease progression which might require a new consultation comforted them in their position without fearing that patients would wonder about an incorrect diagnosis or a superinfection. Finally, some GPs also mentioned that the use of the pad forced them to clarify their diagnosis and to be consistent with it in their prescribing (Quote 12).

#### The patient information leaflet: a redundant tool

About half of the GPs appreciated the educational information of the leaflet but found it redundant with their prescribing order and the explanations they used to give to patients (Quote 13). Filling it was considered fastidious and time-consuming by some GPs and others perceived the leaflet’s objective to be a way—not useful—to convince the patient of the need for an antibiotic treatment. No GP had any idea about the acceptability of the leaflet by patients.

### Patients’ acceptability of the intervention (Table 4)

**Table 4:** Selection of the most illustrative verbatims from patients’ interviews

Subtheme	Quote	Interview number	Verbatim
Appreciation of the intervention and its tools	14	P 10	“I think both are important. I think it is important to have general information. Through the media, or through I don't know, public health organisations. You need to confirm with the GP […] It's especially for people who are resistant to information […] people still trust their GP even if they are stubborn.”
	15	P 6	“They are overbooked […] There are some patients who don’t have a connection with their GP, they just consult him/her to obtain their prescription.”
	16	P 10	“Yes, I think it's [the charter] for any audience. […] It's quite understandable.”
	17	P 2	“Yes, every time they [patients] complain and ask for an antibiotic, with the document they would understand. […] I kept it [the pad] and I even made my children read it”
	18	P 7	“I think it’s [the leaflet] clear, ‘don’t share your antibiotics’, it’s a reflex that people often have. […] It's explicit, the antibiotic, the doses to be taken, the duration of the treatment, I think it's a very well-done document. It also provides information such as the fact that you can return the antibiotics to the pharmacy, honestly I wouldn't have done that if they were already started, I'd have thrown them in the bin. So it gives me information that I think is necessary.”
Perceived impacts on medical care and on their own views towards antibiotics	19	P 2	“People would not go to the GP for a yes or a no, they would understand [the importance of preserving antibiotics] when they see this [the tools]”
	20	P 4	“I think he/she [patient] would not try to say ‘Doctor, I have a flu, give me an antibiotic’.”
	21	P 3	“We’ve all heard about it ‘Antibiotics are not automatic’.”
	22	P 4	“These bacteria [resistant], you can transmit them by saliva, by spitting, by anything, by sneezing. […] And they (my children) are at risk of getting sick because of these bacteria […] I understand it (the information on the pad) like that”

*GP* general practitioner

#### Patients’ appreciation of the intervention and its tools

Almost all patients were in favour of the intervention and only a minority expressed doubts about its effectiveness. Many recognized their GP’s medical authority and trusted him/her, considering that it was his/her responsibility to implement antibiotic stewardship interventions like AntibioCharte (Quote 14). The other patients considered that the GP was not the most appropriate individual/way to educate patients, citing their lack of time to discuss and their lack of authority (Quote 15). They thought that TV campaigns are more effective to convey such messages.

The charter was appreciated by almost all patients, with a content perceived to be clear and understandable (Quote 16). However, most patients acknowledged not having the time or opportunity to read documents in the waiting room.

Most patients appreciated the pad in its form and content. Eleven patients effectively received the pad during the study, and six of them stored it and still have it. All patients considered the pad as a reference document in case of infections for themselves or their relatives (Quote 17). However, some of them expressed doubts about its usefulness if the document is distributed by the GP without explanation.

The leaflet was praised by almost all patients. It was considered useful to make people aware about the specificities of antibiotics and their risks, and to remind them of good practices (Quote 18).

#### Patients’ perceived impacts on medical care and on their own views towards antibiotics

Most patients felt that the use of the tools did not impair the quality of care provided by their GP. Only a minority feared that it could encourage doctors to under-prescribe antibiotics, especially the pad. Most patients believed that the tools (especially the pad and the leaflet) could improve the appropriate use of antibiotics by patients (e.g., by limiting self-medication) (Quotes 19–20).

Patients stated that the intervention confirmed the knowledge about antibiotics they had acquired from previous awareness campaigns (e.g., antibiotics are only needed in case of bacterial infections, could have adverse reactions), and made them aware that viral infections were not always mild (information on symptoms and their duration on the pad) (Quote 21). Even if the majority were aware of the issue of antibiotic resistance before the study, most of them realised thanks to the tools that they had an individual responsibility about it (Quote 22).

## Discussion

AntibioCharte was a randomised controlled study aimed at engaging GPs, facilitating the doctor/patient communication and decreasing patients’ expectations for antibiotics by using three tools made available to GPs during a 1-year period: (1) a commitment charter; (2) a non-prescription pad; and (3) a patient information leaflet. Both GPs and patients found the commitment charter’s content and form relevant, but few GPs felt themselves publicly engaged by the charter. Its location in the waiting room may not be appropriate as patients did not always read the displayed documents, and GPs may forget it. GPs appreciated the non-prescription pad and found that it could help them change their practices. Patients also appreciated it, even if some of them feared than it could encourage doctors not to prescribe antibiotics. Unlike GPs, who considered the patient information leaflet useless and redundant with the information given during the consultation, patients appreciated it.

Few studies evaluated the effectiveness of a commitment charter on physicians’ antibiotic prescribing practices in primary care and they showed contradictory results. Displaying a commitment poster promoting antibiotic stewardship in examination rooms led to a 20% reduction in unnecessary prescriptions for acute respiratory infections in the US [19], but had no effect on antibiotic prescribing in the UK [23]. Our results suggest that the commitment charter of the AntibioCharte study did not fully meet its objectives (i.e., engage GPs in practice improvement and inform patients), perhaps partly because it was displayed in the waiting room. Due to organisational and cultural factors, French physicians are however used to display educational documents/leaflets in the waiting room, where patients generally spend more time than in the examination room. Our results also suggest that mechanisms underlying the public commitment charter’s intended impact (e.g., high value placed on consistency, identification of the prompted behaviour with one’s self-image) [19] may vary according to the socio-cultural context [24].

Patients often do not distinguish between viral and bacterial infections, and believe that the perceived severity of an infection justifies an antibiotic treatment [9, 11, 25, 26]. Besides, patients’ demand for antibiotics (real and/or perceived by doctors) is a well-known reason for unnecessary antibiotic prescribing, especially for respiratory tract infections [10, 27, 28]. Studies from the UK and New Zealand showed that a non-prescription pad may be a useful tool for GPs to educate patients on how to deal with viral infections’ symptoms [29], and can be effective in reducing patients’ expectations to receive antibiotics for colds or flu [14]. In our study, the pad was well appreciated by both GPs and patients. It made patients aware that viral infections can have annoying symptoms (e.g., high fever and long symptoms’ duration), and allowed GPs to fulfil patients’ expectations while avoiding unnecessary antibiotic use. In France, prescription often represents the outcome/end of the consultation, and both patients and physicians value its symbolic [30]. For patients, it shows that the physician listened to her/him, was concerned about her/him, and chose the treatment based on a specific diagnosis. The pad may sometimes represent an alternative to the antibiotic prescription.

To the best of our knowledge, no previous study in primary care has assessed the acceptability or effectiveness on antibiotic use of a document similar to the leaflet. Such a tool, well perceived by patients, may be useful to reduce inappropriate practices concerning antibiotics [31], and may improve knowledge [32]. However, its acceptability by GPs was limited, because they found it redundant with their own prescribing order, and found it time-consuming to complete both documents. An electronic version of the leaflet, automatically produced by the GP’s software, might improve acceptability.

Surprisingly, about half of the interviewed GPs did not perceive themselves as being higher-than-average prescribers of antibiotics, and thus believed that the intervention could only have a limited impact on their practices. Strategies to reduce GPs’ misperceptions about their own practices should be also explored, as they might enhance the impact of antibiotic stewardship interventions.

The AntibioCharte study tested an innovative intervention, relying on three complementary tools targeting both GPs and their patients. This qualitative study explored both GPs’ and patients’ perceptions of the intervention. The random selection of 30 GPs allowed the collection of a variety of opinions and uses of the tools. Evaluation of the GPs’ fidelity in the intervention will help interpreting the results to come assessing the impact of the intervention on antibiotics prescribed by GPs. Data on acceptability of the intervention by the target populations are essential information to decide on the generalisation (or not) of the intervention at regional or national levels in case of significant results. This study has however some limitations. GPs who agreed to participate in the AntibioCharte study, and thus in this qualitative investigation, were probably more interested in antibiotic stewardship and more willing to accept the evaluated tools than those who declined. Patients were mostly recruited by their GP, and lots of them were already concerned about antibiotic resistance. So, caution is needed in extrapolating these results to all French GPs and patients. Finally, we used tools which comply with the French national guidelines and their content may need to be adapted to other national recommendations if applicable.

## Conclusion

The AntibioCharte intervention was well accepted by GPs and patients. The public commitment charter was not really perceived as engaging by GPs. The non-prescription pad showed the most potential for both GPs and patients. The patient information leaflet may improve patient’s knowledge and behaviours about antibiotics, but GPs’ acceptability was limited.

## Supplementary Information

**Additional file 1:** COREQ checklist (Table S1), intervention tools (Documents S1–S3) and interview guides (Documents S4–S5).

## Data Availability

No data are available.

## Abbreviation

GPs: General practitioners
