# Supplementary material for: French general practitioners’ and patients’ acceptability of a public commitment charter and patient information leaflets targeting unnecessary antibiotic use: a qualitative study
Source: Antimicrob Resist Infect Control. 2022 Feb 8;11:32. doi: 10.1186/s13756-022-01065-3 (PMC8822724; doi:10.1186/s13756-022-01065-3)
Supplement: Supplementary file 1 — Additional file 1: COREQ checklist (Table S1), intervention tools (Documents S1–S3) and interview guides (Documents S4–S5). [file 13756_2022_1065_MOESM1_ESM.docx]

**Additional file 1: Table S1. COREQ checklist**

| **No Item** | **Guide questions** | **Reported in page** |
| --- | --- | --- |
| **Domain 1: Research team and reflexivity** | | |
| Personal Characteristics | | |
| Interviewer/facilitator | Which author/s conducted the interview or focus group? | 19 |
| Credentials | Credentials What were the researcher’s credentials? E.g. PhD, MD | 6 |
| Occupation | What was their occupation at the time of the study? | 6 |
| Gender | Was the researcher male or female? | 6, 19 |
| Experience and training | What experience or training did the researcher have? | 6 |
| Relationship with participants | | |
| Relationship established | Was a relationship established prior to study commencement? | 4,5 |
| Participant knowledge of the interviewer | What did the participants know about the researcher? e.g. personal goals, reasons for doing the research | 4,5 |
| Interviewer characteristics | What characteristics were reported about the interviewer/facilitator? e.g. Bias, assumptions, reasons and interests in the research topic | N/A |
| **Domain 2: study design** | | |
| Theoretical framework | | |
| Methodological orientation and Theory | What methodological orientation was stated to underpin the study? e.g. grounded theory, discourse analysis, ethnography, phenomenology, content analysis | 7 |
| Participant selection | | |
| Sampling | How were participants selected? e.g. purposive, convenience, consecutive, snowball | 4,5 |
| Method of approach | How were participants approached? e.g. face-to-face, telephone, mail, email | 5 |
| Sample size | How many participants were in the study? | 7 |
| Non-participation  Setting | How many people refused to participate or dropped out? Reasons? | 7 |
| Setting of data collection | Where was the data collected? e.g. home, clinic, workplace | 7 |
| Presence of non-participants | Was anyone else present besides the participants and researchers? | No |
| Description of sample | What are the important characteristics of the sample? e.g. demographic data, date | 8, 9 |
| Data collection |  |  |
| Interview guide | Were questions, prompts, guides provided by the authors? Was it pilot tested? | 6, 19, S4, S5 |
| Repeat interviews | Were repeat interviews carried out? If yes, how many? | No |
| Audio/visual recording | Did the research use audio or visual recording to collect the data? | 7 |
| Field notes | Were field notes made during and/or after the interview or focus group? | No |
| Duration | What was the duration of the interviews or focus group? | 7 |
| Data saturation | Was data saturation discussed? | 5 |
| Transcripts returned | Were transcripts returned to participants for comment and/or correction? | No |
| **Domain 3: analysis and findings** | | |
| Data analysis | | |
| Number of data coders | How many data coders coded the data? | 7, 19 |
| Description of the coding tree | Did authors provide a description of the coding tree? | No |
| Derivation of themes | Were themes identified in advance or derived from the data? | 7 |
| Software | What software, if applicable, was used to manage the data? | 7 |
| Participant checking  Reporting | Did participants provide feedback on the findings? | No |
| Quotations presented | Were participant quotations presented to illustrate the themes / findings? Was each quotation identified? e.g. participant number | 13, 24 |
| Data and findings consistent | Was there consistency between the data presented and the findings? | 7-15, 24 |
| Clarity of major themes | Were major themes clearly presented in the findings? | 7-15 |
| Clarity of minor themes | Is there a description of diverse cases or discussion of minor themes? | 7-15 |

**Document S1. Commitment charter* used in the AntibioCharte study**


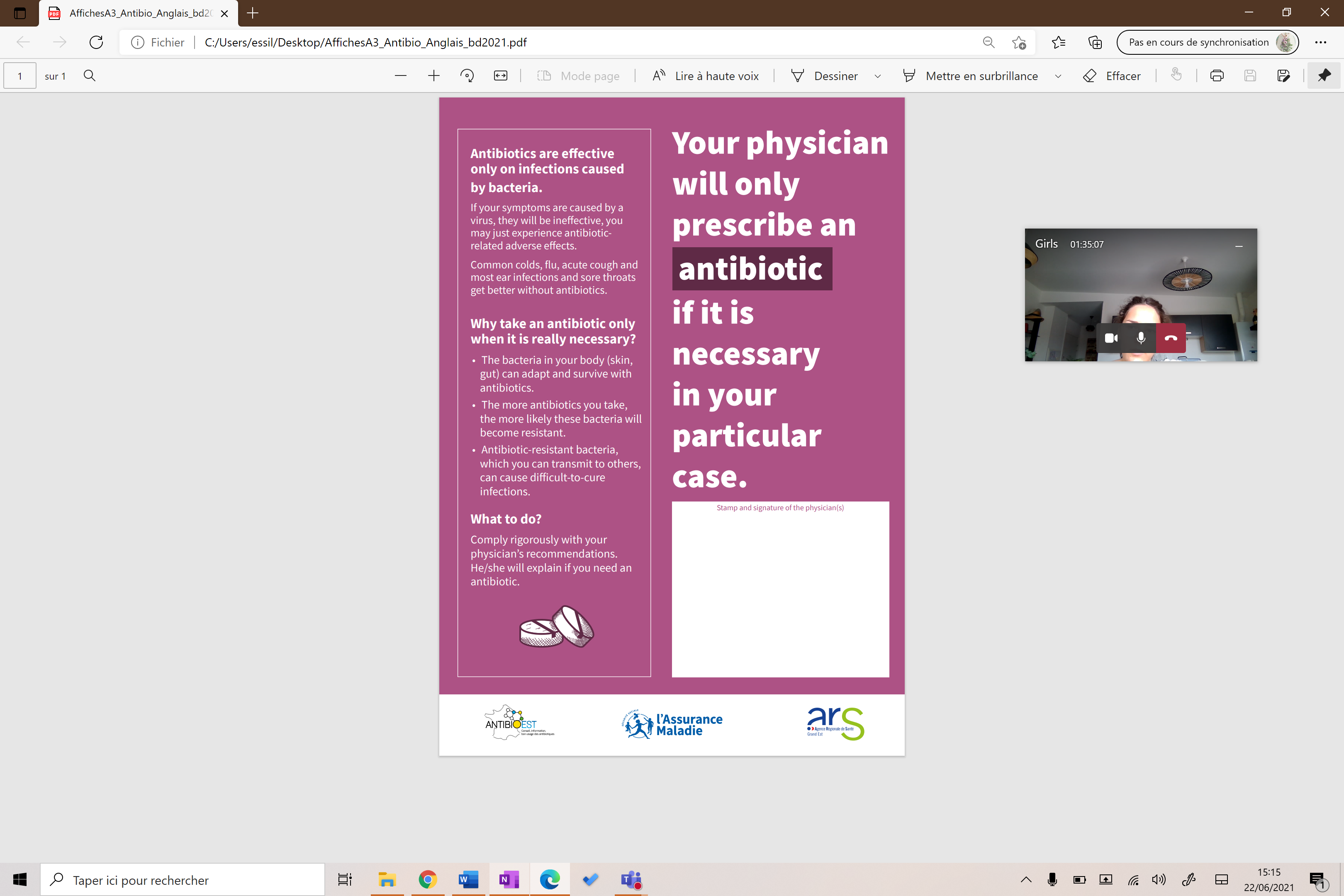


* Translated in English by the authors for the purpose of the publication.

**Document S2. Non-prescription pad* used in the AntibioCharte study**


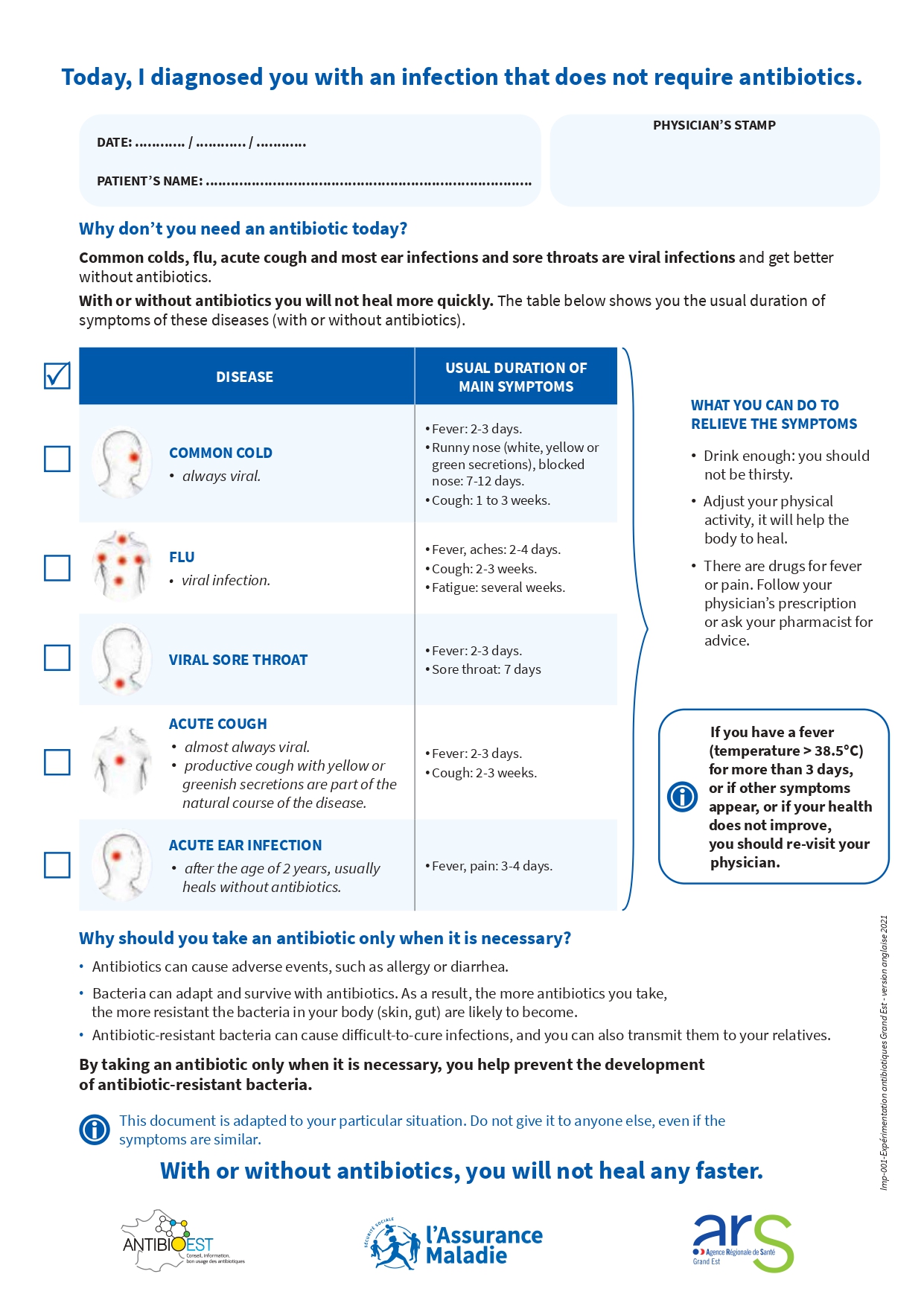


* Translated in English by the authors for the purpose of the publication.

**Document S3. Patient information leaflet* used in the AntibioCharte study**


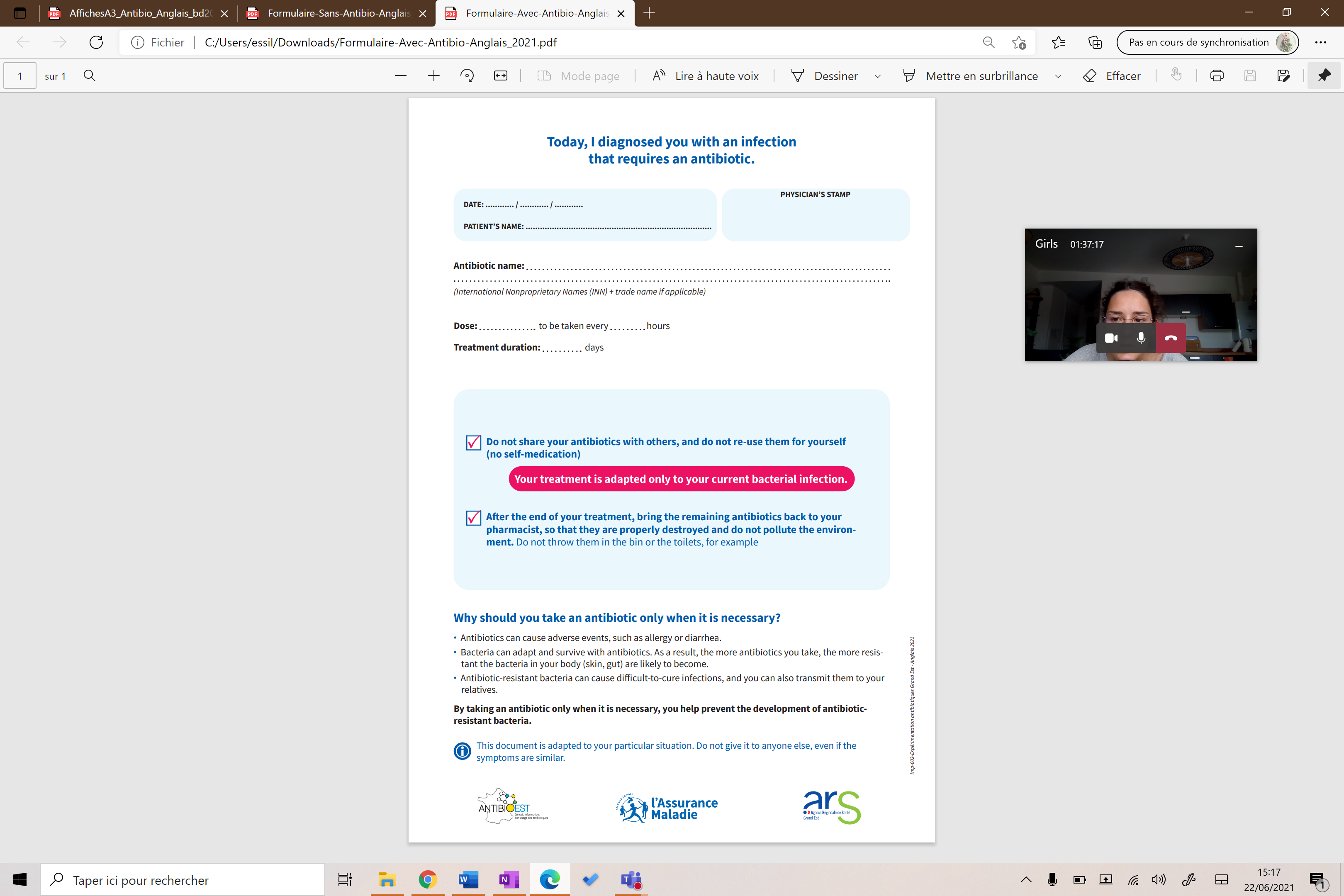


* Translated in English by the authors for the purpose of the publication.

**Document S4. General practitioners’ interview guide**

1. **Did you use the documents? Which ones?**

Follow-up question:

- If yes, how often? How did you use it?
- If not, or if they stopped, why?

1. **What did you think of the documents?**

Follow-up question:

- Pedagogical and clear? If not, which tool and why?
- Negotiating tool?

1. **Do you have examples when they have been particularly useful?**
2. **Did you have any constraint while using the tools? What kind? Did they prevent you using the tools?**

Follow-up question:

- Time constraint?
- Organisational constraint, too energy consuming
- Require too much involvement, new responsibilities

1. **How your patients reacted to the tools?**

Follow-up question:

- Questions, request for explanation
- Astonishment
- Criticism, anger, aggressiveness
- How did you handle those reactions?
- Were they easy to handle?

1. **Do you feel that you have prescribed differently and/or noticed a change in the patients' demand for antibiotics?**

Follow-up question:

- Do you think you have prescribed less antibiotics?
- Do you feel that patient expectations of antibiotics have improved?
- If so, which aspect of the intervention do you think has had the most impact on the evolution of patients’ demand? Which type(s) of patients do you think were particularly impacted? For which infection? If not, why?

1. **Do you feel that the tools have had an impact on the quality of care?**

Follow-up question:

- Do you think that the implementation of the intervention improved the quality of care you delivered?
- Have you been able to treat patients more appropriately for the infections they had?
- Do you think you were encouraged to under-prescribe antibiotics? Did you ever regret not prescribing antibiotics? Have you been blamed for this?

1. **Do you feel that the tools are useful to raise awareness and/or accountability for both GPs and the patients?**

Follow-up question:

- Did you feel (even more) engaged by the display of the charter and/or the use of tools?
- Do you feel that patients behave more responsibly when prescribing antibiotics? What makes you think so (discussion, remarks, questions from patients)?
- In your opinion, which of the components of the intervention seemed to be the most decisive in creating a sense of responsibility in patients?

**Document S5. Patients’ interview guide**

1. **What do you think of the appearance of the three documents?**

Follow-up question:

- Is the Charter visible in the waiting room and does it make you want to read it?
- Images of the non-prescription pad?
- Presentation of the leaflets?

1. **What do you think these documents are for?**

Follow-up question:

- In your opinion, what was the purpose of these documents?

1. **Which document did he/she use with you?**

Follow-up question:

- In which circumstances?
- In your opinion, why?
- What did you feel?
- Did you do anything differently?
- Did you take your antibiotics differently?

1. **How do you feel about your doctor not prescribing or refusing you an antibiotic?**
2. **What do you think of antibiotics?**

Follow-up question:

- In which cases do you think they are necessary?
- In which cases do you think they are not useful?
- Is it a harmless/risk-free treatment, in your opinion?

1. **Have you ever heard of antibiotic resistance?**

Follow-up question:

- If so, what is antibiotic resistance, in your own words?
- Does it worry you? Why or why not?

1. **In your opinion, should measures be taken against antibiotic resistance?**

1. **Did you perceive antibiotics differently after reading the documents?**
2. **Do you think you need to change your behaviour/the way you use antibiotics?**

Follow-up question:

- Why or why not?
- For you and your family?
- What do you plan to do (or not to do)?

1. **Do you think it is necessary to reduce the prescription of antibiotics in general?**

Follow-up question:

- Do you feel personally involved?

1. **Do you think the study/documents have had an impact on the quality of care?**
2. **What do you think should be done to improve the effectiveness of the documents?**
